# Surface Discharges Performance of ETFE- and PTFE-Insulated Wires for Aircraft Applications

**DOI:** 10.3390/ma15051677

**Published:** 2022-02-23

**Authors:** Jordi-Roger Riba, Manuel Moreno-Eguilaz, Tamerlan Ibrayemov, Maxence Boizieau

**Affiliations:** Campus Terrassa, Universitat Politècnica de Catalunya, 08222 Terrassa, Spain; manuel.moreno.eguilaz@upc.edu (M.M.-E.); ibrayemov.tamerlan@gmail.com (T.I.); maxence.boizieau@reseau.eseo.fr (M.B.)

**Keywords:** aircraft power systems, low-pressure, solar blind sensors, ultraviolet radiation

## Abstract

Compared to their predecessors, the next generations of aircrafts will be more electrified, require more electrical power and operate at higher voltage levels to meet strict weight and volume constraints. The combined effect of low-pressure environments, increased voltage levels and compact designs intensifies the risks of premature insulation degradation due to electrical discharge activity. This paper studies the resistance to surface discharges of PTFE (polytetrafluoroethylene) and ETFE (ethylene tetrafluoroethylene), two insulation materials widely used in today’s aircraft wiring systems due to their outstanding properties, such as a wide temperature operation range and a high dielectric strength. The study is carried out in a low-pressure chamber, which was pressurized within the pressure range of 10–100 kPa that includes most aircraft applications. There is a compelling need for experimental data to assess the resistance of insulation materials to surface discharges at a very early stage as a function of the environmental pressure. Data on resistance to surface discharges in low-pressure environments for aeronautical applications are lacking, while most standards for insulation systems are based on tests under standard pressure conditions. The results provided in this work can be useful to design wiring systems for future more electric aircrafts, as well as to design fault detection systems for an early detection and identification of faults related to surface discharges. Therefore, the data and analysis included in this paper could be of great interest to design and develop insulation systems for wiring systems and standard assessment methods, as well as to design fault detection strategies for the early detection and identification of surface discharges for future generations of more electric aircrafts.

## 1. Introduction

Distribution voltage levels will increase on new aircraft models due to their progressive electrification as a result of recent developments in more electric aircrafts (MEA) and all electric aircrafts (AEA). The combined effect of higher voltages, compact designs and extreme environmental conditions found in next-generation aircrafts pose insulation systems in a challenging position. Operation at higher voltages in combination with the extreme environmental conditions typical of aircraft systems greatly increases the risk of electrical discharge occurrence [1]. As derived from Paschen′s law, low-pressure operation significantly lowers the dielectric strength of atmospheric air [2,3,4]; thus, the surface discharges initiate at lower voltages compared to those found in ground operations. This effect has detrimental consequences, such as insulation degradation, reduced insulation life or complete insulation breakdown [5]. Electrical discharges in wire insulation materials tend to spread along the wires, with the consequent fire hazard [6,7]. Insulation breakdown can lead to catastrophic consequences, including disconnection of circuits, fire, aborted operations, emergency landings or fatal accidents.

Commercial airliners typically fly at altitudes in the range of 9.5 to 11.5 km, although they can climb up to 15 km [5], and most military aircrafts can fly above these altitudes. This wide altitude range implies a wide range of temperatures; therefore, the corresponding pressure range is between 100% and 12% of atmospheric pressure at sea level. At these altitudes, environmental conditions are extreme [8], and different aircraft systems operate in unpressurized areas. Thus, insulation materials in aircraft systems are subjected to very demanding conditions due to higher electric stresses resulting from the combined effect of higher voltage levels, compact form factors, and extreme environmental conditions [9,10].

According to the IEC 60112:2020 standard [11], the tracking phenomenon consists of a progressive development of conductive paths on the surface or/and inside a solid insulating material, due to the combined effects of electrolytic contamination and electric stress. Arc tracking in wires initiates in the form of small surface discharges that pyrolyze the insulation layer of the wire, creating a partly conductive pathway that promotes discontinuous low-magnitude surface discharges, which overheat the insulation [12] and cause more damage. Due to the low-energy surface discharges produced by arc-tracking activity, detection of this phenomenon at a very early stage is not easy [13]. Although the aviation industry is constantly improving fault detection systems, wiring-related faults often remain undetected, so there is an urgent need to develop detection systems and standard procedures to assess the performance of insulation materials in low-pressure environments, and to develop procedures to identify surface discharge activity at a very early stage, before irreversible damage to insulation systems occurs.

Although there are different international standards to determine the resistance of solid electric insulation materials to electric discharges [11,14,15,16,17,18,19], most of the tests described in these standards are performed at standard atmospheric pressure. For example, the European standards EN 3475-604:2018 [18] and EN 3475-603:2018 [19] detail methods for evaluating the behavior of cable insulation when an electric arc is originated and sustained by two energized cables rubbing against a blade in dry conditions [18] and initiated and maintained in the presence of a contaminating fluid along the outer surface of the insulation. These standards aim at produce and control sustained failure effects representative of those occurring in service when conductors are damaged by abrasion (EN 3475-604:2018) and subjected to contamination by aqueous fluid (EN 3475-603:2018). They also aim at examining the aptitude of the insulation against the tracking and propagation of electric arcs, although the tests are performed at standard atmospheric pressure.

By using field-grading materials in the insulation layer or by adding insulating filler compounds, it is possible to reduce the electric stress at the expense of adding complexity, weight, increasing the diameter of the cable [20,21], and worsening the thermal behavior of the cable [5]. Therefore, a proper selection of insulation materials for wiring systems is a critical decision. Aircraft insulation materials in wiring systems basically include polytetrafluoroethylene (PTFE), ethylene tetrafluoroethylene (ETFE), and polyimide (Kapton^®^) [21]. However, polyimides are no longer used in new aircrafts due to the rapid degradation they undergo when exposed to the combined effect of moisture, heat, and mechanical stress [21,22].

This work studies the surface discharge behavior of PTFE and ETFE insulation materials commonly used in current wire insulation aircrafts under 400 Hz in the pressure range of 10–100 kPa, which is representative of most aircraft applications. It also presents a low-pressure test bed designed to qualify wire insulation materials under this pressure range to simulate the operating environment of unpressurized areas of aircrafts. The experimental results presented in this work allow a fair comparison of the performance of the two insulation materials studied and show the vital role that pressure plays in the inception of surface discharges. Due to the development of MEA aircrafts, there is an urgent need to generate experimental data to design compact wire insulation systems as well as to develop test systems to assess the resistance of insulation materials to surface discharges, taking into account the variable pressure range found in aircraft systems. Currently, there is a scarcity of experimental data related to the resistance of wire insulation materials to surface discharges under low-pressure conditions found in aircrafts, in part because most standard tests for qualifying insulation systems are performed under standard pressure conditions. Therefore, it is necessary to understand and address the role of wire insulation materials for preventing surface discharge occurrence, in order to optimize the reliability of insulation systems operating at low pressure. The results derived from this work may be valuable not only for designing wiring insulation systems for next-generation MEA aircrafts, but also for developing failure detection methods for the early detection of surface discharge activity.

The organization of this document is as follows. Section 2 details the experimental setup, including the applied sensing method, the wire electrodes, and the low-pressure chamber used to generate the surface discharges, as well as the high-voltage source. Section 3 describes the experiments carried out with the PTFE- and ETFE-insulated wire samples and compares the results attained. To conclude, Section 4 develops the conclusions of this paper.

## 2. Experimental Setup to Compare the Arc Tracking Performance of PTFE and ETFE Insulations in Low-Pressure Operation

This section details the components of the experimental setup, including the sensor used to detect the UV radiation emitted by surface discharges, the wire electrodes and insulation materials used, as well as the components of the low-pressure chamber used for the tests performed under different pressures and the variable high-voltage source.

### 2.1. The Sensor Used to Detect the Surface Discharges

Before complete breakdown, electrical discharges produce partial discharges, which in air gaps are perceived as corona discharges [23] in the most stressed areas [24,25]. Partial discharges can be detected using expensive devices, including UHF detectors [26], partial discharge detectors and radio interference voltage devices [27], acoustic noise meters [28,29], or spectrophotometers [30], among others. Discharge activity can also be detected by optical methods [31], including visible and ultraviolet (UV) sensors. Some of these instruments are very sensitive, compact, have a reasonable cost, and allow the detection of electrical discharges at a very early stage [3]. However, in daylight conditions, the optical radiation emitted by electrical discharges can be interfered by sunlight, since it includes, among others, UV and visible wavelengths, this being a serious drawback of most optical sensors.

The UV spectrum includes wavelengths in the range of 100–400 nm, which is divided into UVC (100–280 nm), UVB (280–315 nm), and UVA (315–400 nm) spectral ranges. It is an accepted fact that ozone in the stratosphere absorbs most of the extraterrestrial radiation that falls within the UVB and UVC ranges. Solar blind sensors allow one to avoid the interference of sunlight, so they fall in the category of optical sensors that can only detect UV radiation with wavelengths less than 280 nm [32]. Thus, solar blind sensors are only sensitive to UV radiation with wavelengths shorter than those of the solar spectrum after being filtered by the Earth’s atmosphere.

Due to the abovementioned characteristics, the solar blind R9533 UVTRON sensor (Hamamatsu Photonics, Hamamatsu City, Japan) was used to detect discharge activity at a very early stage due to different relevant features, including small size, very reduced power consumption, high sensitivity, pressure range compatible with the requirements of this work and reasonable cost. This sensor incorporates a gas-filled tube inside, in which an electron avalanche multiplication phenomenon occurs due to a high-voltage difference applied between the photocathode (negative electrode) and the anode (positive electrode).

The main features of the gas-filled tube UV-sensitive sensor used in this work are summarized in Table 1.

To optimize the performance of the R9533 UVTRON sensor, a suitable driver circuit is required. In this way, by applying a low-voltage to the driver circuit, the sensor can operate safely. In addition, the signal processing circuitry built into the driver circuit cancels out sporadic background noise, thereby minimizing the probability of false detection events. To this end, the commercial C10807 (Hamamatsu Photonics, Hamamatsu City, Japan) driver circuit was used to supply the R9533 sensor. Table 2 shows some of the features of this driver circuit.

Previous internal tests were carried out in the AMBER laboratory of the Universitat Politècnica de Catalunya. In such tests, the performance and sensitivity of the solar blind R9533 UVTRON sensor was compared with that of a single-loop antenna sensor and a back-illuminated CMOS imaging sensor. The results verified the sensitivity and accuracy of the UV-sensitive solar blind sensor in detecting UV radiation emitted by the discharges at a very early stage [23].

### 2.2. The Notched Wire Electrodes Used in the Experiments

**Figure 1:**
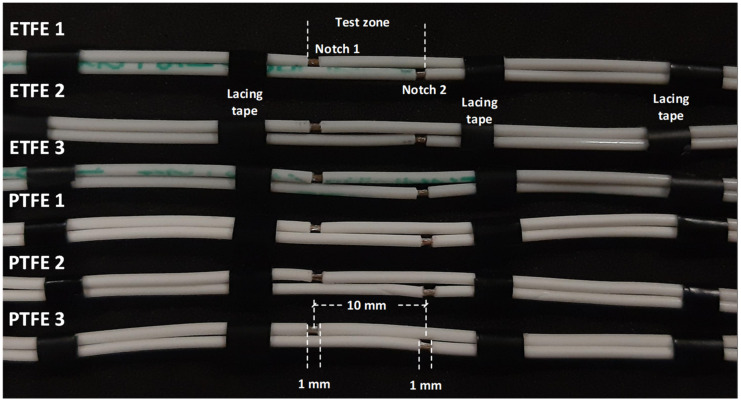
ETFE- and PTFE-insulated pairs of wire samples analyzed in this work. A notch made in each wire sample, 1 mm wide and 10 mm apart from the notch of the mating wire. The mating wires were fastened using lacing tapes to ensure the wires are straight and parallel, while ensuring continuous contact within the test zone.

This section describes the specific electrodes used to determine the performance to surface discharges of PTFE- and ETFE-insulated wires. To this end, wire electrodes are used to reproduce in the laboratory the surface discharges occurring in real aircraft environments. It should be noted that this paper focuses on detecting surface discharges at a very early stage, long before significant consequences can arise. The wire electrodes were damaged following the procedure detailed in the European standard EN 3475-603:2018 [19], which describes methods to assess the behavior of cable insulation when an electric arc is created and sustained between energized wires. The electrodes consist of two artificially damaged parallel wires; the conductive core of one of the wires is connected to the high-voltage electrode, while the core of the other wire is grounded. The analyzed pairs of wire samples are approximately length 0.5 m long each, and the insulation layer on their ends is stripped to allow electrical connection to the high-voltage source and ground, respectively. The two lengths of the cable were artificially damaged by cutting the insulation layer around the full circumference of the insulation, ensuring that the cut penetrates the conductor around the full circumference. A 1.0 mm wide portion of the insulation was then removed, as shown in Figure 1. Before the tests, the length of each wire was cleaned using a clean cloth moistened with isopropyl alcohol.

**Figure 2:**
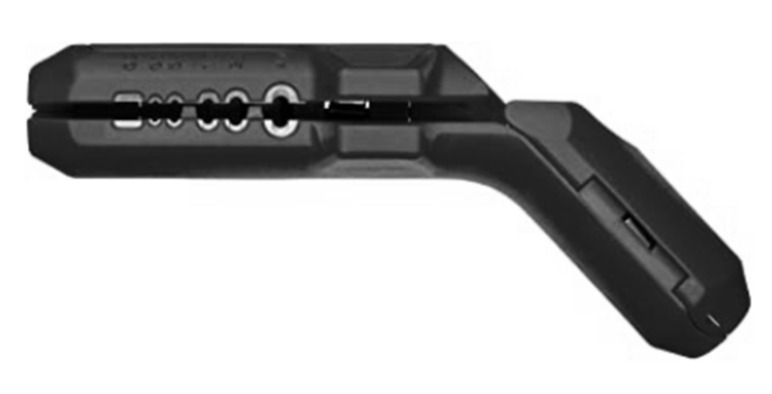
Cable stripping tool used to artificially damage the analyzed wires.

A cable-stripping tool (KNIPEX ErgoStrip, Wuppertal, Germany) was used to cut the insulation layer, which is shown in Figure 2.

In order to compare the arc tracking performance of various specimens, it is very important to ensure that the cuts made on the different specimens are as similar as possible. To this end, the width of the cuts and the distance between them were measured with the help of digital photographs. Using a 54 Mpixels digital camera (approximately 9000 × 6000 effective pixels), resolutions of around 10 micrometers can be achieved. Even so, as it is not possible to perform identical notches in the insulation layer, three replicas of the pairs of insulated wire samples were made for each type of insulation; these replicas allowed for the evaluation of the dispersion of the results.

Table 3 shows the main properties of the analyzed PTFE- and ETFE-insulated wires.

It is also noted that both analyzed wires have the same size (AWG 24) and insulation thickness, so the results can be compared.

Table 4 shows some physical and electrical properties of PTFE and ETFE materials.

### 2.3. The Low-Pressure Chamber

As the experiments presented in this work are realized in a wide pressure range that covers the 10–100 kPa interval, a low-pressure chamber is required. For this purpose, a 375 mm × 260 mm (height × diameter) cylindrical stainless-steel container was used, with a methacrylate lid sealed with a silicon rubber gasket to prevent air from entering the chamber from outside. This chamber also includes two vacuum-tight access ports, the first one for the high-voltage cable and the second one for the wires that supply the solar blind UV-sensitive sensor.

**Figure 3:**
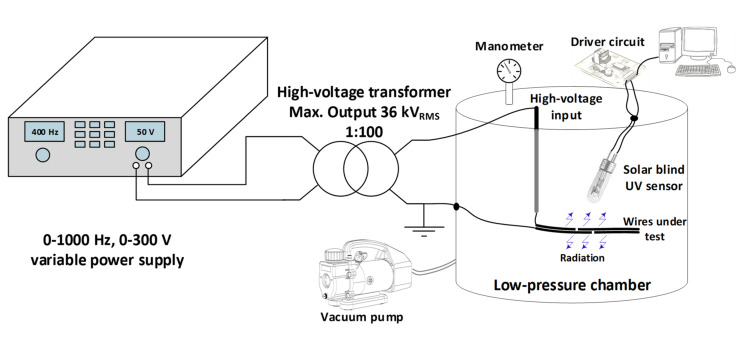
Sketch of the experimental layout used to detect the UV light emitted by the electrical discharges in the very initial stage using a solar blind UV sensor in the 10–100 kPa range using a low-pressure chamber connected to the high-voltage transformer and the vacuum pump.

A vacuum pump is required to vary the pressure inside the low-pressure chamber within the pressure range of 10–100 kPa. To this end, a single-stage BA-1 vacuum pump (1/4 HP, 0.085 m^3^/min manufactured by Bacoeng, Suzhou, Jiangsu, China) was used to reduce the pressure in the low-pressure chamber. The pressure inside the low-pressure chamber was measured with an analog manometer integrated with the chamber. The experiments were carried out at a temperature of 25 °C and the humidity was limited below 25%. More details of the low-pressure chamber can be observed in Figure 3.

### 2.4. The High-Voltage Variable Supply

To determine the CEV values under different pressure levels, a variable high-voltage power supply is required. To this end, a low-voltage power supply was combined with a single-phase high-voltage transformer. To obtain a variable high-voltage in the range of 0–36 kV, a SP300VAC600W power supply from APM Technologies (0–300 V, ±0.1 V, 0–1000 Hz, Dongguan, China) was linked to a VKPE-36 single-phase high-voltage transformer manufactured by Laboratorio Electrotécnico (max. output voltage 36 kV, turns ratio = 100, Cornellà de Llobregat, Spain). It should be noted that during the tests, the output frequency of the SP300VAC600W power supply was adjusted to 400 Hz, the characteristic distribution frequency in current aircrafts. As the SP300VAC600W power supply has a sensitivity of ±0.1 V and the high-voltage transformer steps up the voltage by a factor of 100, the minimum voltage step on the high-voltage side is 10 V.

Figure 3 describes the experimental arrangement used to reduce the pressure inside the low-pressure chamber in order to apply a variable high-voltage to the two wire electrodes and to measure the UV radiation emitted during the initial stage of the surface discharges.

## 3. Experimental Results

The results presented in this section have been obtained using the experimental setup described in Figure 3 and the mating wire samples shown in Figure 1.

The experimental data presented in this section are based on the corona extinction voltage (CEV), i.e., the minimum value of the voltage at which corona activity (UV radiation emission) is detected with the solar blind sensor. To determine the CEV value at each pressure level, the following procedure is applied. First, the voltage is gradually increased starting from 0 kV until the solar blind sensor detects corona activity, this being the corona inception voltage. The voltage is then further increased by approximately 10%. Next, the voltage is gradually reduced until the solar blind sensor detects no UV radiation coming from the analyzed mating wire electrodes. The minimum voltage at which UV radiation is detected corresponds to the CEV value. It is worth noting that because under CEV conditions, i.e., at the very early stage of discharge activity, due to the very low energy level of discharges and noisy aircraft environments, the analysis of UV emissions is very convenient. Otherwise it is very complex to detect the phenomenon by measuring electrical signals.

### 3.1. Results Obtained with PTFE Samples

This section presents the CEV values obtained with the PTFE-insulated wires described in Section 2.2 in the pressure range of 10–100 kPa.

**Figure 4:**
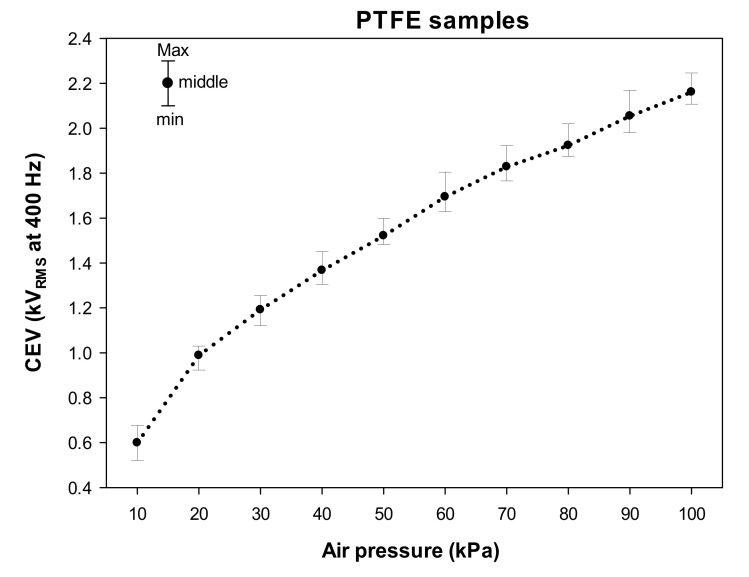
CEV values measured at 400 Hz of the analyzed PTFE samples (three replicas each) in the 10–100 kPa interval covering the aeronautic pressure range.

Figure 4 shows the experimental CEV values measured within the 10–100 kPa pressure range in steps of 10 kPa using the solar blind gas-filled sensor. The error graphs show the values of the three analyzed samples for each pressure point (minimum, middle, and maximum values).

The results presented in Figure 4 clearly show a decline of CEV values when the pressure is reduced. This is because the mean free path of electrons decreases with increasing air pressure, so more collisions occur in the path of electrons between the two electrodes. Collisions reduce the energy of electrons and make it more difficult for a molecule to ionize.

### 3.2. Results Obtained with ETFE Samples

**Figure 5:**
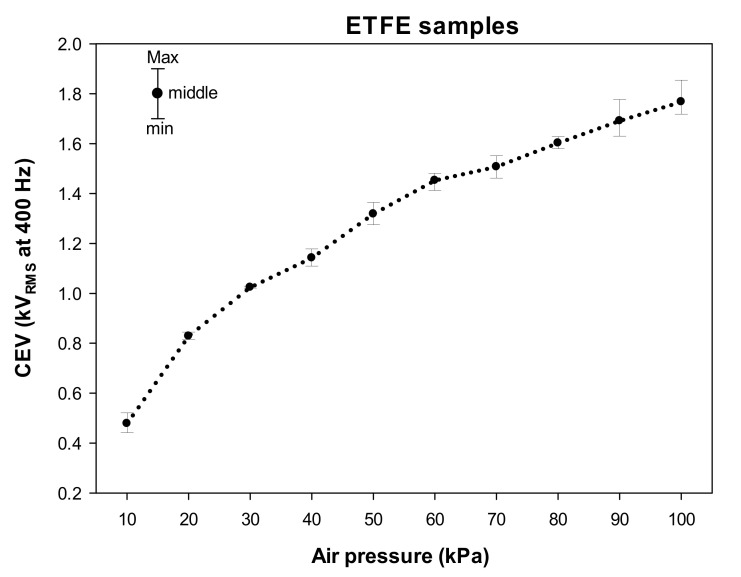
CEV values measured at 400 Hz of the analyzed ETFE samples (three replicas each) in the 10–100 kPa interval covering the aeronautic pressure range.

Figure 5 shows the CEV values measured within the pressure range of 10–100 kPa in steps of 10 kPa using the solar blind gas-filled sensor. The error graphs show the values of the three analyzed samples for each pressure point (minimum, middle, and maximum values).

Once again, the results presented in Figure 5 show an evident reduction of the CEV values with atmospheric pressure, due to the increase in the mean free path of the electrons when the pressure of air is reduced, which allows the electrons to accelerate at higher speeds, producing more energetic collisions, thus facilitating ionization processes.

### 3.3. Comparative Results with PTFE and ETFE Wire Samples

**Figure 6:**
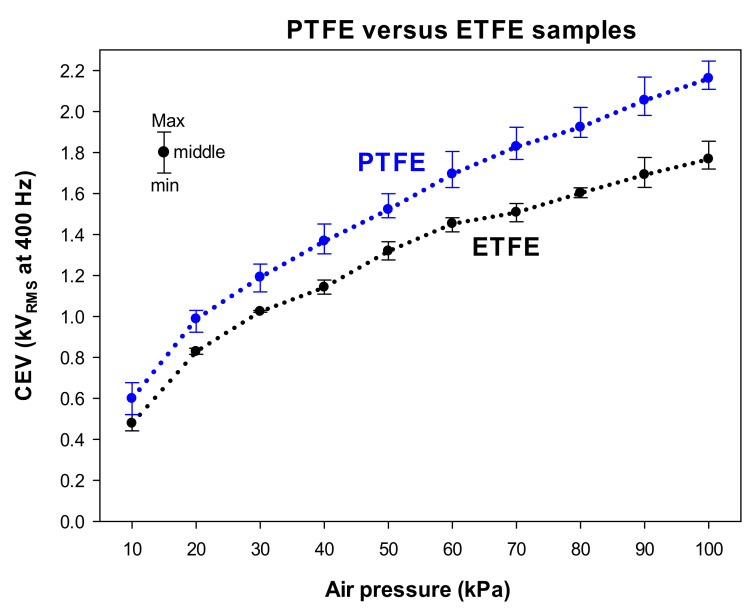
Comparative CEV values measured at 400 Hz of the analyzed PTFE and ETFE samples (three replicas each) in the 10–100 kPa interval covering the aeronautic pressure range.

Figure 6 compares the experimental CEV values in the pressure range of 10–100 kPa achieved with the PTFE- and ETFE-mating wire electrodes.

The comparative results presented in Figure 6 show a similar trend of the CEV-pressure curves for the analyzed PTFE and ETFE samples. The curves in Figure 6 clearly show a better performance of the PTFE insulation compared to ETFE, as the CEV values of PTFE-insulated wires are always higher than those of ETFE-insulated wires. These results clearly indicate that PTFE-insulated wires withstand higher voltages before surface discharges initiate. These results are in line with previous results reported in [33], which indicate that PTFE insulation requires higher voltages to initiate surface discharges compared to ETFE. However, in [33], the CEV values are not studied and the effect of pressure is not analyzed in depth.

For a better interpretation of the results presented in Figure 6, Table 5 displays the average values of the CEV for both types of wire samples, as well as the relative differences of the CEV values at the different pressures covering the 10–100 kPa pressure range.

The results presented in Table 5 prove that in the pressure range of 10–100 kPa, AWG 24 PTFE-insulated wires withstand on average 16.45% more voltage before surface discharges initiate. This effect may be due to the higher surface resistivity and lower dielectric constant of PTFE compared to ETFE (see Table 4). It is worth noting that the higher value of the dielectric constant of ETFE insulation compared to PTFE causes a higher electric stress in the air surrounding the sample, which favors the inception of discharges.

Table 6 shows the average percent reduction in CEV values with pressure for both types of wire samples.

The results presented in Table 6 show a similar trend of reduction of CEV values with pressure for both types of analyzed insulation materials.

## 4. Conclusions

Future aircraft designs will be more electrified, requiring more electrical power and higher distribution voltage levels to meet stringent weight and volume constraints. The combination of low-pressure conditions, higher voltage levels, and dense form factors in next-generation aircrafts increases the risks of premature insulation degradation due to surface discharges.

This paper has investigated the resistance to surface discharges of two insulation materials intended for aircraft wiring systems, namely PTFE and ETFE. For this, artificially damaged insulated wire electrodes have been used, so that surface discharges can be effectively replicated on the different electrodes and analyzed and detected at a very early stage. The discharges have been detected using a small-size low-cost solar blind UV sensor, which detects the UV radiation emitted by the discharge activity without any interference from sunlight. The analyzed PTFE- and ETFE-insulated wires have been tested in a low-pressure chamber in the pressure range between 10 kPa and 100 kPa, which accounts for most aircraft applications. The experimental data presented in this work show that, under the conditions of this study, PTFE insulation outperforms ETFE in terms of resistance to surface discharges, because the average value of CEV (corona extinction voltage) of PTFE-insulated wires is 16.5% higher than that of ETFE-insulated wires. The results provided in this work can be useful to design wiring systems for future more electric aircrafts, as well as to design fault detection systems for early detection and identification of faults related to surface discharges.

## Figures and Tables

**Table 1:** Hamamatsu R9533 UVTRON sensor used to detect the UV radiation emitted by the electrical discharges.

Parameters	Values
Type	Gas-filled tube
Main application area	Flame sensor
Spectral range	185–260 nm (solar blind)
Direct current supply voltage	350 V ± 25 V
Average discharge current	0.3 mA (max. 1 mA)
Estimated life	25 × 10^3^ h
Allowable operating temperature	−40–125 °C
Background noise	<10 counts/min
Sensitivity (typical value)	10^4^ counts/min
Weight	2.5 g

**Table 2:** Hamamatsu C10807 driver circuit used to supply the UV sensor.

Parameters	Values
Output voltage	350 V
Supply voltage	12–24 V
Current consumption	4 mA
Estimated life	25 × 10^3^ h
Operating temperature	−10–50 °C
Operating humidity	<80%
Sensitivity (typical value)	10^4^ counts/min
Dimensions	50 mm × 12 mm × 36 mm

**Table 3:** Main characteristics of the analyzed PTFE- and ETFE-insulated wires.

Properties	Values/Description	
	PTFE	ETFE
Manufacturer	AlphaWire	Thermax
Size	AWG 24	AWG 24
Strands	7/32	19/36
Applicable standards	AWM/STYLE 1213 MIL-W-16878/4 (Type E)	MIL-W-22759/16 MIL-W-22759/17
Insulation material	PTFE	Extruded ETFE
External diameter	1.12 mm	1.09 mm
Insulation thickness	0.25 mm	0.24 mm
Temperature interval	−60–200 °C	−55–150 °C
Rating	600 V_RMS_	600 V_RMS_

AWG—American Wire Gauge.

**Table 4:** Properties of PTFE and ETFE materials.

Parameters	PTFE	Extruded ETFE
Specific gravity (ASTM D792)	2.2 g/cm^3^	1.74 g/cm^3^
Volume resistivity (ASTM D1531)	>10^18^ Ohm cm	>10^16^ Ohm cm
Surface resistivity (ASTM D257)	>10^18^ Ohm	>10^16^ Ohm
Dielectric constant (ASTM D1531, 1 MHz)	2.1	2.6
Arc resistance (ASTM D495)	>300 s	122 s
Dielectric strength (ASTM D149)	24 kV/mm (2.01 mm)	15 kV/mm (3.2 mm)

**Table 5:** CEV average values difference (%) between PTFE and ETFE samples at the different analyzed pressures.

Pressure (kPa)	PTFE Average Value (kV)	ETFE Average Value (kV)	Difference (%)
10	0.599	0.478	20.20%
20	0.988	0.828	16.19%
30	1.191	1.024	13.99%
40	1.367	1.142	16.46%
50	1.521	1.319	13.30%
60	1.694	1.453	14.25%
70	1.828	1.508	17.52%
80	1.923	1.602	16.68%
90	2.054	1.691	17.66%
100	2.161	1.768	18.20%
		**Average difference**	16.45%

**Table 6:** CEV values reduction (%) as a function of the pressure with respect to the value at 100 kPa.

Pressure (kPa)	PTFE	ETFE
10	72.28%	72.96%
20	54.28%	53.16%
30	44.89%	42.05%
40	36.74%	35.40%
50	29.62%	25.40%
60	21.61%	17.82%
70	15.41%	14.71%
80	11.01%	9.35%
90	4.95%	4.32%
100	0.00%	0.00%

## Data Availability

Not applicable.
